# CNT:TiO_2_-Doped Spiro-MeOTAD/Selenium Foam Heterojunction for High-Stability Self-Powered Broadband Photodetector

**DOI:** 10.3390/nano15120916

**Published:** 2025-06-12

**Authors:** Yuxin Huang, Pengfan Li, Xuewei Yu, Shiliang Feng, Yanfeng Jiang, Pingping Yu

**Affiliations:** School of Integrated Circuits, Jiangnan University, Wuxi 214122, China; 6231916005@stu.jiangnan.edu.cn (Y.H.); 6231916037@stu.jiangnan.edu.cn (P.L.); 6231916052@stu.jiangnan.edu.cn (X.Y.); 6201924073@stu.jiangnan.edu.cn (S.F.); jiangyf@jiangnan.edu.cn (Y.J.)

**Keywords:** photodetector, heterojunction, Se, Spiro-MeOTAD

## Abstract

Photodetectors are critical components in modern optoelectronic systems due to their extensive applications in information conversion and image storage. Selenium (Se), an element with a low melting point, a broad spectral response, and rapid response speed, exhibits a disadvantage of high optical reflectivity, which leads to a reduction in response. Spiro-MeOTAD, featuring controllable energy bands and facile processing, has its practical application limited by inadequate thermal and environmental stability. In this study, Spiro-MeOTAD-1 with enhanced stability was prepared through the optimization of dopants (Zn(TFSI)_2_ and CNT:TiO_2_) within Spiro-MeOTAD, to create a Se-F/Spiro-MeOTAD-1 heterojunction photodetector by subsequently compositing with selenium foam (Se-F). The self-powered device demonstrates exceptional photovoltaic performance within the wavelength range of 350–800 nm at 0 V bias, exhibiting a maximum responsivity of 108 mA W^−1^, a switching ratio of 5 × 10^3^, a specific detectivity of 2.96 × 10^12^ Jones, and a response time of 20 ms/50 ms. The device also demonstrates elevated environmental stability pretreatment at 140 °C following a one-month period. The photodetection stability of the Se-F/Spiro-MeOTAD-1 flexible PD was demonstrated by its capacity to retain 76.3% of its initial light current when subjected to 70 bending cycles at 30°. This finding further substantiates the photodetection stability of the material under various bending conditions. Further verification of the applicability of Spiro-MeOTAD-1 in Se-based devices establishes a novel paradigm for designing photodetectors with enhanced performance and stability.

## 1. Introduction

Operating through direct photoelectric conversion, self-powered photodetectors (PDs) achieve optical signal detection without requiring external power input. Self-powered photodetectors play an important role in the fields of communication, imaging, and environmental monitoring [1,2,3]. Traditional inorganic p-type semiconductors for photodetectors, such as silicon (Si) [4], nickel oxide (NiO) [5], and copper oxide (CuO) [6], are still widely used due to their mature processes and stability. However, their narrow bandgap and low carrier mobility limit performance improvement. Organic p-type semiconductors, such as P3HT and PEDOT:PSS [7,8], have the advantages of flexibility and low cost, but their carrier mobility is relatively low and needs to be optimized through molecular design (such as introducing conjugated groups). By constructing p-n junctions, Schottky junctions, or van der Waals heterojunctions, the built-in electric field is enhanced to improve the separation efficiency of photogenerated carriers [9,10,11]. Therefore, to produce heterojunctions with good self-powered photoelectric capabilities and broadened wavelength responses, it is critical to choose p-type organic and inorganic semiconductors with high light absorption coefficients and adjustable band gaps [12,13].

Selenium (Se), as an intrinsic p-type semiconductor, has a low melting point (217 °C), broad spectral response (UV-visible), and fast response, making it an ideal material for photodetectors [14,15,16,17,18]. Se structures including nanowires, microtubes, nanoflowers, and films can be prepared using physical vapor deposition, chemical vapor deposition, and liquid phase exfoliation [19,20,21]. With a highest responsivity of 19 mA W^−1^ upon 610 nm, the single Se microtube photodetector exhibits broadband responsivity in the UV-Vis range [22]. However, the strong optical reflectivity of Se materials causes low responsivity. The p-n Se/ZnO heterojunction PD exhibits switching ratios of four orders of magnitude and a response time of within 0.5 s at 0 V and 370 nm (0.85 mW cm^−2^) illumination [23]. The heterojunction structures composed of organic (PANI, PEDOT, PPy, Spiro-MeOTAD) and inorganic (RGO, CsPbBr_3_, and ZnO) semiconductors deposited on the Se micro–nano structure also exhibit excellent performance with self-powered characteristics [24,25,26,27]. This suggests that Se can be utilized in heterojunction-type photodetectors to extend the range of optical response. Selenium encapsulated in an N-doped porous carbon framework foam was used as an electrode material in a lithium–selenium battery, aimed at enhancing the long-term cycling performances [28]. A Se-TiO_2_ cathode coating on a nickel foam for lithium–selenium batteries exhibits a reversible discharge capacity of 600.4 mAh g^−1^ at 0.5 C after 200 cycles [29]. Depositing Se onto a foam structure substrate improves electrochemical performances. Consequently, creating a Se foam with a unique nanoporous structure directly simplifies the overall design of photodetectors and enhances their excellent photovoltaic properties, eliminating the need for substrate optimization. Therefore, the hole transport efficiency of selenium-based detectors is constrained by the inherent conductivity of the material, and there is an urgent need to break through the performance bottleneck through the design of new Se foam heterojunctions and interface optimization [30,31,32].

Organic semiconductor materials (e.g., Spiro-MeOTAD) have the advantages of tunable energy bands, easy processing, and flexibility, which can effectively increase light absorption and reduce reflection losses. Spiro-MeOTAD is mainly used as a hole transport layer (HTL) in solar cells, and is now also gradually being applied in photodetectors. It has been demonstrated to form heterojunctions with inorganic semiconductors such as Ga_2_O_3_ [33], TiO_2_ [34], and Sb_2_S_3_ [35] to successfully construct heterojunction-type photodetectors with good optoelectronic performance. Huang et al. reported that n-Calcite/p-Spiro-MeOTAD heterojunction devices exhibited a remarkable photovoltaic conversion efficiency of 17.64% and excellent photodetection characteristics under AM 1.5 G illumination with a 0.01 V driving voltage. The devices were characterized by a 0.17 s short response time, and high switching repeatability and stability [36]. Yan et al. presented a photodetector with a p-i-n structure (p-Spiro-MeOTAD/Ga_2_O_3_/n-Si). The structure can work without any external power supply under the effect of dual internal electric fields. Spiro-MeOTAD thickness was found to have an impact on the optoelectronic performances, with a peak responsivity of 192 mA W^−1^ at 2 μW cm^−2^ at zero bias and 254 nm illumination [36]. However, its thermal and environmental stability is poor, especially in high temperatures, and its air environment performance is easy to degrade. To solve the above problems, the enhancement of the stability of Spiro-MeOTAD through a Zn(TFSI)_2_ complex doping mechanism was reported by Saygili et al., which can achieve efficient oxidation under oxygen-free and light conditions. It was revealed that the coordination of zinc ions with tert-butylpyridine (tBP) resulted in the formation of stable [Zn(tBP)_3_]^+^ (TFSI^−^) complexes (ΔE = −1.52 eV), which significantly enhanced the stability of oxidized state Spiro-MeOTAD^+^ [37,38,39]. It is shown that Zn(TFSI)_2_ can drive the oxidation in a dark environment (with a significant increase in the intensity at the absorption peak 523 nm), and the reversibility of the oxidized state after light exposure is superior compared to the conventional LiTFSI dopant. XPS and FT-IR confirmed the strong coordination between zinc and tBP, which effectively suppressed the ionic migration, and the device showed a remarkable enhancement in thermal properties and stability. It provides a new strategy for the doping mechanism of organic semiconductors, which has important potential for application in chalcogenide solar cells and other optoelectronic devices [40,41,42].

**Figure 1:**
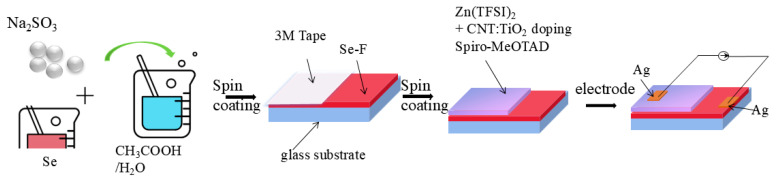
An illustration of the Se-F/Spiro-MeOTAD-1 heterojunction preparation procedure.

In this study, the Spiro-MeOTAD-1 was prepared by optimizing dopants with Zn(TFSI)_2_ and CNT:TiO_2_ composite-doped Spiro-MeOTAD to improve stability and optoelectronic properties. Selenium foam (Se-F) was prepared by the solution method, showing a porous structure to increase the effective light absorption area and flexibility. High-performance heterojunction photodetectors were constructed by compounding Se-F with optimized Spiro-MeOTAD-1 (Figure 1), combining the advantages of broad spectral absorption, efficient charge separation, and fast transport. The porous structure of selenium extends the light-trapping capability, while the double doping strategy significantly enhances the stability and conductivity of the hole transport layer. Energy band matching at the heterojunction interface suppresses carrier complexation while broadening the optical response range of the device. The device exhibits excellent self-powered characteristics, high responsivity (108 mA W^−1^), and a fast response speed (20 ms/50 ms) over a wide spectral range (368 nm–876 nm). In addition, the stability of the device in extreme environments is significantly improved, laying the foundation for practical applications. This design presents a new method for creating inexpensive, high-performing photodetectors with a wide range of possible applications in integrated optoelectronics and flexible electronics.

## 2. Materials and Methods

Preparation of Se-F: The preparation of Se-F is mainly divided into aqueous solution deposition and annealing crystallization. A total of 200 mg of high-purity Se powder (Aladdin, 99.99%) and 1.4 g of Na_2_SO_3_ (Aladdin, 99.99%) crystal particles (mass ratio Se:Na_2_SO_3_ = 1:7) was added into 50 mL of deionized water and stirred at 90 °C for 12 h to ensure that the Se powder was completely dissolved and generated Na_2_SeO_3_ following Equation (1). An amount of 2 mL of glacial acetic acid (CH_3_COOH) (TCI, 99.5%) was added to the above solution, and Na_2_SeO_3_ was reacted to form monomer Se with the reaction formula as in Equation (2):

(1) Na_2_SO_3_ + Se → Na_2_SeSO_3_

(2) Na_2_SeSO_3_ + 2CH_3_COOH → Se↓ + 2CH_3_COONa + SO_2_↑ + H_2_O

A 1 × 1 cm glass substrate was placed in the bottom of the beaker and used to deposit the Se red monomers in a refrigerator for about 5 h. After being repeatedly cleaned with anhydrous ethanol and deionized water, the thick red a-Se film was allowed to dry at ambient temperature. The dense red a-Se film was placed in a tube furnace with nitrogen gas and heated to 190 °C at a rate of 3 °C min^−1^ for 1 h, resulting in the Se-F with higher crystallinity.

For the preparation of Spiro-MeOTAD-1, 72.3 mg of Spiro-MeOTAD powder (Sigma-Aldrich, 95%) was dissolved in 1 mL of chlorobenzene. Then, 28.8 μL of 4-tert-butylpyridine (tBP) (Sigma-Aldrich, 98%) was added dropwise to the chlorobenzene (Sinopharm Shanghai Trial, AR, 99.5%) solution containing Spiro-MeOTAD powder, followed by the addition of 17.5 μL of a mixed solution of acetonitrile (Sinopharm Shanghai Trial, AR, 99.5%) and Zn(TFSI)_2_ (Sinopharm Shanghai Trial, AR, 99.5%), where the Zn(TFSI)_2_ powder was 400 mg and anhydrous acetonitrile was 1 mL (mass concentration of 400 mg mL^−1^). A total of 19 μL of CNT:TiO_2_ (TCI, 95%) dissolved in chloroform (Sinopharm Shanghai Trial, AR, 99.5%) (concentration 0.08 mg mL^−1^) was added to the above mixed solution to obtain the optimized Spiro-MeOTAD-1 solution. The chloroform action was mainly to eliminate the recessed holes on the membrane surface.

Se-F/Spiro-MeOTAD-1 device: One side of the Se-F was covered with a 3M tape. The other side of the Se-F was covered with a Spiro-MeOTAD-1 layer by a spin-coating process to obtain the effective Se-F/Spiro-MeOTAD-1 composite area. Ag paste is used as the electrode to prepare the Se-F/Spiro-MeOTAD-1 device. Under the same conditions, Se-F/Spiro-MeOTAD devices can be obtained by selecting Spiro-MeOTAD for spin-coating.

A Raman spectrometer (LabRam1B, 632.8 nm, Renishaw Confocal Raman Spectrometer, UK), an X-ray diffractometer (XRD, BrukerD8-A25, Bruker AXS GMBH, German), a scanning electron microscope (SEM, JEOLJSM-7000F, JEOL, Janpan), and a UV-Vis absorption spectrometer (VarianCary 500, Varian, USA) were used to analyze the morphologies and structures of the samples. Photoluminescence (PL) measurements were obtained by an FLS-980 D2D2 system, accompanied by excitation sources of 532 nm to facilitate the analysis of the optical characteristics. X-ray photoelectron spectra (XPS) and ultraviolet photoelectron spectra (UPS) were obtained using a Shimadzu AXISULTRADLD-600W (KRATOS, UK) device. Using a semiconductor test system based on the Keithley 4200 (Keithley, USA), the optoelectronic characteristics of the devices were described.

## 3. Results

**Figure 2:**
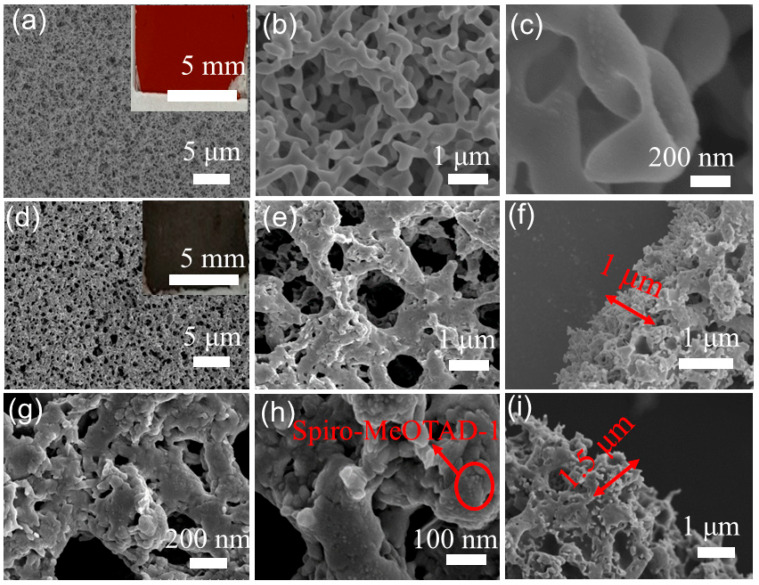
(**a**–**c**) SEM images of a-Se and (**d**,**e**) Se-F at different magnifications, and their corresponding optical graphs in (**a**,**c**). The SEM image of Se-F cross-section (**f**). (**g**–**h**) SEM images of Se-F/Spiro-MeOTAD-1, and the cross-section SEM image (**i**).

The dense a-Se film is a red color (Figure 2a), showing a porous mesh interwoven structure composed of irregularly bifurcated rods (Figure 2b). The surface of the a-Se rods is not smooth, exhibiting protruding and concave structures, with a diameter of approximately 300 nm (Figure 2c). The a-Se membrane was calcined at 190 °C to form the Se-F with a foam-like structure with the distribution of large and small mesh pores with different diameters ranging from 200 nm to 900 nm (Figure 2d). Upon closer inspection with higher magnification, the enlarged mesh pore structure reveals a similar laminar structure in its interior (Figure 2e). The thickness of the Se-F is about 1 μm as shown in Figure 2f. The diameter of Se-F/Spiro-MeOTAD-1 decreases in Figure 2g, maintaining a rod-like structure. The Spiro-MeOTAD-1 solution adhered well to the walls of the Se-F surfaces, showing dotted bumps of different sizes (Figure 2h). In Figure 2i, the thickness of the Se-F/Spiro-MeOTAD-1 film is approximately 1.5 μm.

**Figure 3:**
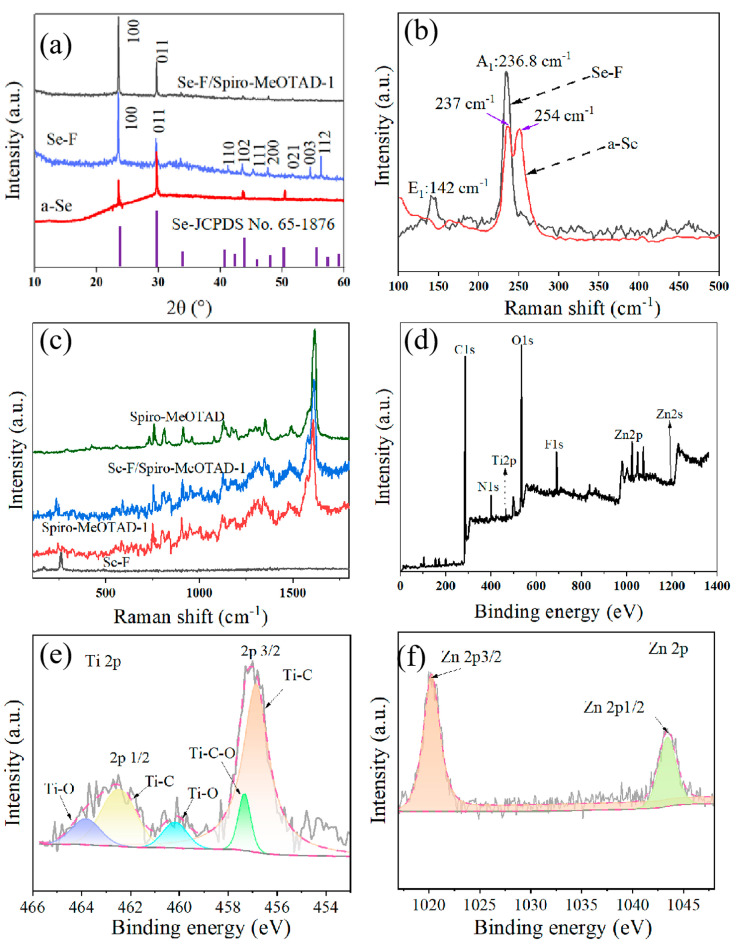
XRD patterns of a-Se, Se-F, and Se-F/Spiro-MeOTAD-1 (**a**), Raman spectra of Se-F and a-Se (**b**), Raman spectra of Se-F, Spiro-MeOTAD, Spiro-MeOTAD-1, and Se-F/Spiro-MeOTAD-1 (**c**), XPS spectrum of Spiro-MeOTAD-1 (**d**), and high-resolution survey spectrum of (**e**) Ti 2p and (**f**) Zn 2p.

The XRD patterns of Se-F and a-Se films exhibit the same diffraction peaks but the intensity of the diffraction peaks is generally higher compared to that of the a-Se film (Figure 3a). The high intensity of the (100) crystalline plane of Se-F suggests higher crystallinity consistent with JCPDS No. 65-1876, and more annealing than that of a-Se [24,25,26]. The high intensity peaks observed for a-Se at 142 cm^−1^, 237 cm^−1^, and 254 cm^−1^ in Figure 3b are in good agreement with the transverse photoacoustic E1 mode, with an amorphous selenium bonding structure (Se8 ring) [9,20,43]. The peak at 236.8 cm^−1^ attributed to Se-F corresponds to the characteristic A_1_ stretching mode of chained t-Se, indicating that a modification in selenium bonding has occurred following annealing, which leads to a distinct Raman resonance peak. Figure 3c shows the Raman spectra of Se-F, Spiro-MeOTAD, Spiro-MeOTAD-1, and Se-F/Spiro-MeOTAD-1. The Spiro-MeOTAD spectra exhibit peaks at 1616, 1175, 1075.8, 1211.7, and 1479 cm^−1^, which correspond to the benzene ring C-C vibrations, the bending of the C-H quinoline ring [21], the vibration of the S=O polar bond, the stretching vibration of the C-Cl bond in chlorobenzene, and the stretching of C=N in the quinoline ring. The different peaks at 590 cm^−1^ and 250 cm^−1^ of Spiro-MeOTAD-1 relate to the symmetric stretching mode of the Ti-O bond of TiO_2_ and the transverse vibration of Zn with respect to N in the N-Zn-N chain. The presence of characteristic peaks of Se-F and Spiro-MeOTAD-1 in the Se-F/Spiro-MeOTAD-1 spectra confirms the successful formation of the heterojunction [44]. The chemical composition and state of Spiro-MeOTAD-1 were analyzed by XPS and are shown in Figure 3d. The detected elements include C, O, N, F, and Zn, with the Ti and Zn peak originating from TiO_2_ and Zn(TFSI)_2_. The Ti 2p binding energies of Spiro-MeOTAD-1 are positioned at 462.6 eV and 456.9 eV, which are ascribed to the 2p1/2 and 2p3/2 orbital levels, respectively (Figure 3e). The peaks at 457.3 eV, 458.9 eV, and 463.8 eV represent the characteristic Ti-O and Ti-C-O bonds of TiO_2_ doped in Spiro-MeOTAD [42]. The deconvoluted 2p peaks at 462.4 eV and 456.8 eV reveal Ti-C bonds of Ti 2p1/2 and 2p3/2 orbitals, respectively. The Zn 2p high-resolution survey spectrum of Spiro-MeOTAD-1 clearly presents two major peaks centered at 1020.5 eV and 1043.6 eV with a spin-energy difference of 23 eV, corresponding to Zn 2p1/2 and Zn 2p3/2, respectively (Figure 3f) [41]. The elemental compositions derived from the analysis are consistent with those introduced by the added chemicals.

**Figure 4:**
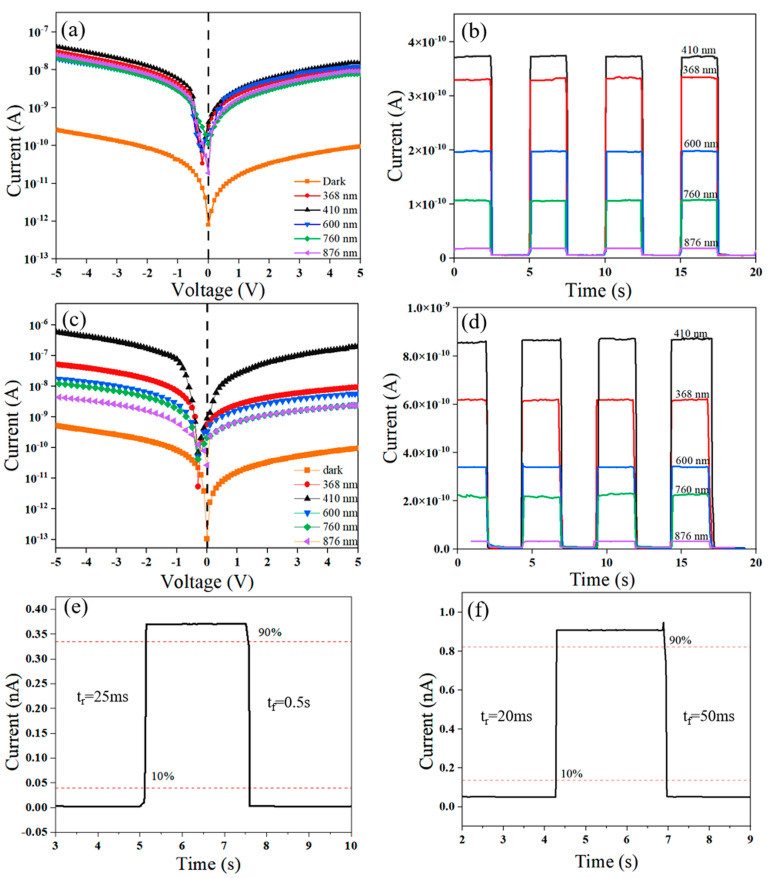
(**a**) I-V and (**b**) I-t curves of Se-F/Spiro-MeOTAD PD in dark and at 368–876 nm. (**c**) I-V and (**d**) I-t curves of Se-F/Spiro-MeOTAD-1 PD in dark and at 368–876 nm. (**e**) Single-cycle I-t curve of Se-F/Spiro-MeOTAD at 410 nm. (**f**) Single-cycle I-t curve of Se-F/Spiro-MeOTAD-1 PD at 0 V bias.

The I-V curves of the Se-F/Spiro-MeOTAD device at different wavelengths (368–876 nm) are shown in Figure 4a. The dark current (I_d_) is 9.62 × 10^−13^ A and the minimum value of the light current (I_light_) occurs at a voltage of −0.25 V [45], indicating that the Se-F/Spiro-MeOTAD PD exhibits an excellent self-powered characteristic. The forward bias current is significantly larger than the reverse bias current, demonstrating that the device possesses a rectification ratio of about 10 ± 1. At 0 V bias, the Se-F/Spiro-MeOTAD PD at 410 nm exhibits a maximum light current of about 3.73 × 10^−10^ A with a switching ratio of 388, while the minimum light current is 2.34 × 10^−11^ A at 876 nm (Figure 4b). Compared to the Se-F/Spiro-MeOTAD PD, the dark current of the Se-F/Spiro-MeOTAD-1 PD is much smaller, about 1.46 × 10^−13^ A (Figure 4c). The lowest point of the logarithmic curve is V_oc_ = 0.3 V with a very good self-powered characteristic [46,47]. The photocurrent response of the Se-F/Spiro-MeOTAD-1 photodetector (PD) across the spectral range from 350 nm to 900 nm under 0 bias is larger than that at the same wavelength in the Se-F/Spiro-MeOTAD; in particular, at 410 nm it is increased by 12.8 times with a switching ratio of 5 × 10^3^ as shown in Figure 4d. The rising and falling times of the Se-F/Spiro-MeOTAD PD and Se-F/Spiro-MeOTAD-1 PD are 25 ms/50 ms (Figure 4e) and 20 ms/50 ms (Figure 4f), respectively. The separation efficiency of the built-in potential for photogenerated carriers is enhanced under light illumination. Additionally, the Spiro-MeOTAD-1 PD exhibits superior hole transport capability, which not only effectively boosts the photocurrent but also enhances the response speed. No significant attenuation of the photocurrent was observed for either the Se-F/Spiro-MeOTAD or the Se-F/Spiro-MeOTAD-1 photodetector (PD) after 100 cycles of photoperiodic irradiation (Figure S1), indicating a good stability.

**Figure 5:**
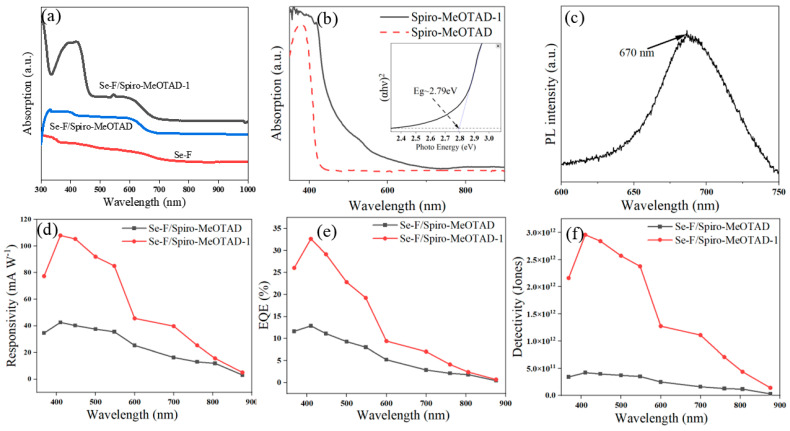
(**a**) UV-Vis absorption spectra of Se-F/Spiro-MeOTAD, Se-F/Spiro-MeOTAD-1, and Se-F. (**b**) UV-Vis absorption and bandgap (Eg) of Spiro-MeOTAD and Spiro-MeOTAD-1. (**c**) PL spectrum of Se-F. (**d**) Responsivity of Se-F/Spiro-MeOTAD and Se-F/Spiro-MeOTAD-1 devices. (**e**) External quantum efficiency of Se-F/Spiro-MeOTAD and Se-F/Spiro-MeOTAD-1 devices. (**f**) Specific detectivity of Se-F/Spiro-MeOTAD and Se-F/Spiro-MeOTAD-1 devices.

The UV-Vis absorption spectrum of Se-F shows significant absorption values across the 300–700 nm wavelength range (Figure 5a), with an absorption cutoff edge around 667 nm, which is consistent with the maximum photoluminescence spectrum (PL) peak in Figure 5c. Compared with Se-F, the light absorption intensities of Se-F/Spiro-MeOTAD and Se-F/Spiro-MeOTAD-1 are improved overall in the extent of 350–1000 nm, with the most significant improvement in the scope of 350–450 nm for Spiro-MeOTAD-1 (Figure 5a). The decrease in absorption intensity at 450 nm corresponds to the absorption cutoff region of Spiro-MeOTAD-1, indicating that the addition of Spiro-MeOTAD-1 is well-suited for enhancing the light absorption of the Se-F/Spiro-MeOTAD-1 heterojunction in the range of 350–450 nm. Throughout the 350–900 nm wavelength range, Spiro-MeOTAD-1 exhibits a higher absorption coefficient than Spiro-MeOTAD (Figure 5b), especially in the 450–550 nm region. The bandgap of Spiro-MeOTAD-1 was calculated to be approximately 2.79 eV. Based on the power densities of incident light at different wavelengths (368 nm of 0.056 mW cm^−2^, 410 nm of 0.049 mW cm^−2^, 600 nm of 0.044 mW cm^−2^, 760 nm of 0.049 mW cm^−2^, and 876 nm of 0.024 mW cm^−2^), the power densities of the Se-F/Spiro-MeOTAD and Se-F/Spiro-MeOTAD-1 devices with responsivity (R_λ_) and specific detectivity (D*) curves are calculated by equations (Supporting Information Note S1 and the effective area of devices in Figure S2).

The responsivity of the Se-F/Spiro-MeOTAD-1 device at 410 nm is approximately 108 mA W^−1^ (Figure 5d), which is 2.5 times higher than that of the Se-F/Spiro-MeOTAD device (42.6 mA W^−1^). The most significant increase in responsivity is observed within the 400 nm to 450 nm wavelength range, as the Spiro-MeOTAD-1 exhibits the largest enhancement of the optical absorption coefficient in this range. The value of external quantum efficiency is proportional to the responsivity and inversely proportional to the range of 350–750 nm. The Se-F/Spiro-MeOTAD-1 PD has a maximum external quantum efficiency of 32.65%, which is an increase of 253.6% compared to Se-F/Spiro-MeOTAD (12.87%) as shown in Figure 5e. The Se-F/Spiro-MeOTAD-1 PD exhibits an EQE enhancement of 0.16% at 806 nm and 0.03% at 876 nm, respectively, compared to the Se-F/Spiro-MeOTAD PD. This enhancement is primarily due to the low responsivity in this wavelength range, resulting in an almost negligible increase in external quantum efficiency. Compared to the Se-F/Spiro-MeOTAD PD (with a maximum D* of 3 × 10^11^ Jones at 410 nm), the Se-F/Spiro-MeOTAD-1 PD demonstrates a higher maximum D* of approximately 2.96 × 10^12^ Jones at 410 nm and a lower minimum of 1.4 × 10^11^ Jones at 876 nm (Figure 5f). The responsivity values of the Se-F/Spiro-MeOTAD PD and the Se-F/Spiro-MeOTAD-1 PD are 42 ± 5 mA W^−1^ and 109 ± 5 mA W^−1^, respectively (Figure S3). The trapping coefficient (C_trap_) of the Se-F/Spiro-MeOTAD-1 PD is 0.92, which achieves only 7.96% of the theoretical limit (ideal C_trap_ of 11.56 using the equations in Supporting Information Note S2, and the thickness of devices is 60 μm as shown in Figure S4), and it is necessary to optimize light-trapping structures in the future. As listed in Table 1, the prepared Se-F/Spiro-MeOTAD-1 PD exhibits self-powered properties with a high on/off ratio, responsivity, and specific detectivity.

**Figure 6:**
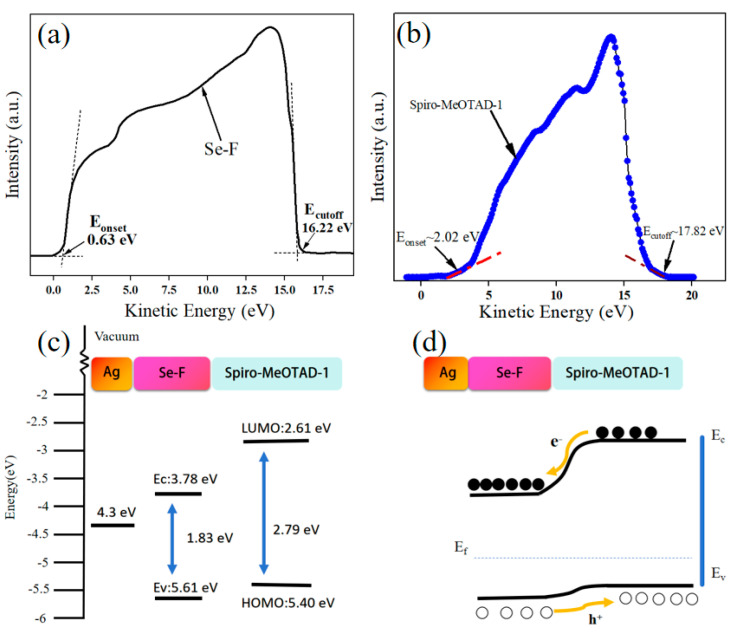
UPS spectra of Se-F (**a**) and Spiro-MeOTAD-1 (**b**). Se-F/Spiro-MeOTAD-1 energy band diagram before (**c**) and after contact (**d**).

Based on the UPS spectra in Figure 6a, the valence band (E_V_) of Se-F is −5.61 eV, as determined by the formula E_V_ = 21.20 − (E_cutoff_ − E_onset_), and the conduction band (E_C_) of Se-F can be calculated to be −3.78 eV, according to the bandgap (E_g_) of 1.83 eV. The E_g_ of Spiro-MeOTAD-1 is calculated to be approximately 2.79 eV in Figure 6b, where its Highest Occupied Molecular Orbital (HOMO) is at −5.4 eV and its Lowest Unoccupied Molecular Orbital (LUMO) is at −2.61 eV. Spiro-MeOTAD-1 and Se-F form a type-II heterojunction-type photodetector as presented in Figure 6c. Under light illumination, the photogenerated electrons are propelled by the inherent electric field to migrate from the high potential LUMO of Spiro-MeOTAD-1 to the lower potential E_C_ of Se-F. In parallel, the photogenerated holes are directed from the low potential E_V_ of Se-F to the higher potential HOMO of Spiro-MeOTAD-1, heading towards the electrodes, which leads to a continuous current (Figure 6d).

**Figure 7:**
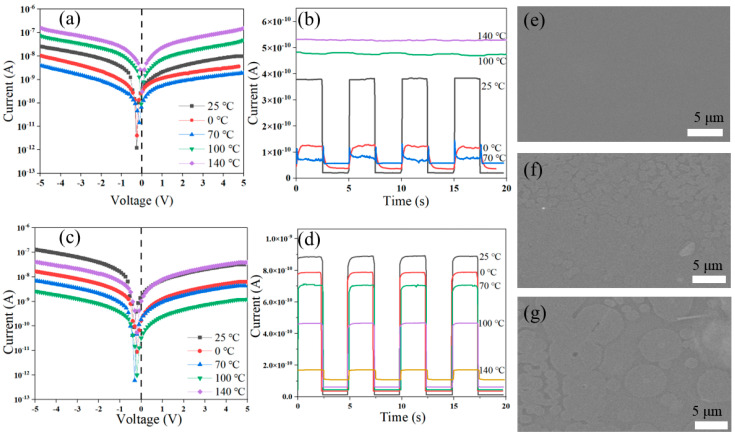
I-V (**a**) and I-t curves (**b**) of Se-F/Spiro-MeOTAD PD at 0 °C, 25 °C, 70 °C, 100 °C, and 140 °C. I-V (**c**) and I-t curves (**d**) of Se-F/Spiro-MeOTAD-1 PD at the same temperature conditions at 410 nm. SEM images of Spiro-MeOTAD-1 film at (**e**) 25 °C, (**f**) 100 °C, and (**g**) 140 °C.

Figure 7a,b are the I-V and I-t curves of the Se-F/Spiro-MeOTAD PD tested under 410 nm illumination with different temperature pretreatments, respectively. As the temperature increases, the built-in potential decreases. At 25 °C, the built-in potential reaches its maximum of 0.25 V, which corresponds to the optimal rectification characteristics. However, higher temperatures lead to a decline in the optoelectronic properties of Spiro-MeOTAD, reducing the intrinsic potential generated by the device. The built-in potential is approximately 0.06 V at 100 °C, and the light current curves for both forward and reverse bias voltages are identical at 140 °C, with nearly no built-in potential. Due to the carrier freezing effect, the light current and the dark current at 0 °C are 1.25 × 10^−10^ A and 4.89 × 10^−12^ A, respectively, and the response time increases to 0.72 s/0.32 s. The light current decreases as the temperature increases; the value at 70 °C is only 6.3% of that at 25 °C. Even the light current and the dark current at 100 °C are almost equal, having increased to about 490 pA. Figure 7c,d show the I-V and I-t curves of Se-F/Spiro-MeOTAD-1 PD after pretreatment with different temperatures at 410 nm under 0 V bias. The built-in potential at 25 °C and 140 °C is 0.3 V and 0.2 V. The maximum photocurrent is reached at 25 °C with the minimized dark current. Due to the high temperature weakening the reverse cutoff property of the device, the dark currents corresponding to 70 °C, 100 °C, and 140 °C are larger than those at room temperature, which are 5.53 × 10^−12^ A, 1.08 × 10^−11^ A, and 2.57 × 10^−11^ A, respectively. The high temperature weakens the hole transporting ability of Spiro-MeOTAD-1 to reduce the photocurrent. The decrease in response speed (50.6 ms/75.2 ms) at 140 °C was not significant. The response speed and photocurrent of the device decreases with increasing temperature due to the scattering effect of the internal lattice of Se at high temperatures which makes the carrier mobility decrease. Spiro-MeOTAD-1 forms a heterojunction detector with significantly improved temperature characteristics compared to Spiro-MeOTAD. The surface of the Spiro-MeOTAD-1 film at 25 °C is smooth and shows good isotropy (Figure 7e). Crystallization occurs on the surface following treatment at 100 °C, yet the surface remains relatively flat and continuous (Figure 7f). Agglomerations of large particles appear after the treatment at 140 °C (Figure 7g). Spiro-MeOTAD-1 does not exhibit dry cracking at high temperatures, possesses superior surface integrity, and demonstrates enhanced optoelectronic properties at elevated temperatures.

**Figure 8:**
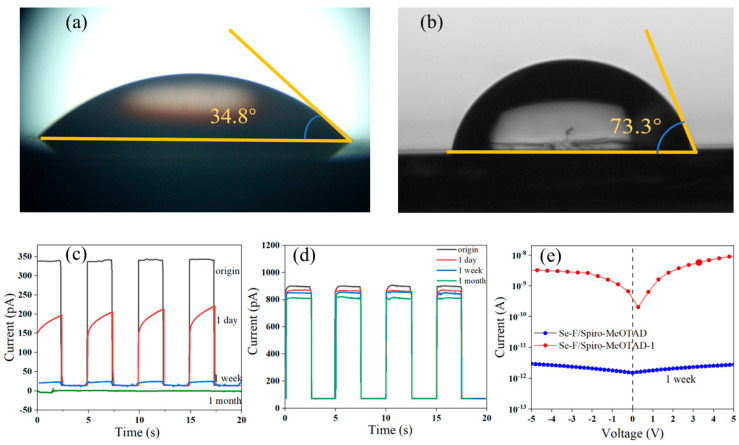
Water contact angles of (**a**) Spiro-MeOTAD and (**b**) Spiro-MeOTAD-1. I-t curves of Se-F/Spiro-MeOTAD (**c**) and Se-F/Spiro-MeOTAD-1 (**d**) devices after different times of placement at 410 nm and 0 V. (**e**) I-V curves of Se-F/Spiro-MeOTAD PD and Se-F/Spiro-MeOTAD-1 PD after 1 week under the same irradiation conditions.

The water contact angle of Spiro-MeOTAD-1 and the Spiro-MeOTAD film is 73.3° (Figure 8a) and 34.8° (Figure 8b), respectively. This indicates that the higher hydrophobicity of Spiro-MeOTAD-1 effectively prevents water molecules from penetrating the film, thereby increasing the optoelectronic performances of the heterojunction photodetectors. The Se-F/Spiro-MeOTAD PD still has stable switching cycles after one day with 57% I_light_ of the original value and a higher I_d_ of 0.94 pA, but the response is slowed down, with the rise and fall times of 70.69 ms/50.22 ms (Figure 8c). After one week, the light intensity of the Se-F/Spiro-MeOTAD PD is approximately 36 pA, the current (I_d_) decreases to 0.82 pA, and the response time is extended by nearly a factor of twelve. The decrease in I_light_ and Id over an extended placement time is attributed to the degradation of hole transport performance resulting from moisture absorption and the redistribution of additives in the Se-F/Spiro-MeOTAD device. The I_light_ values of the Se-F/Spiro-MeOTAD-1 device after one day, one week, and one month are 99.1%, 98.2%, and 88.9% of the original current, respectively (Figure 8d). Figure 8e shows the I-V curves of the Se-F/Spiro-MeOTAD and Se-F/Spiro-MeOTAD-1 devices after one week at 410 nm and 0 V. The Se-F/Spiro-MeOTAD-1 PD still exhibits good rectification and self-powered performance, but the Se-F/Spiro-MeOTAD PD exhibits a smaller light current and lacks rectification capability due to moisture absorption, which results in increased resistance of Spiro-MeOTAD. Therefore, the Spiro-MeOTAD-1 PD can maintain excellent photoelectric properties in an atmospheric environment and exhibits enhanced environmental stability.

**Figure 9:**
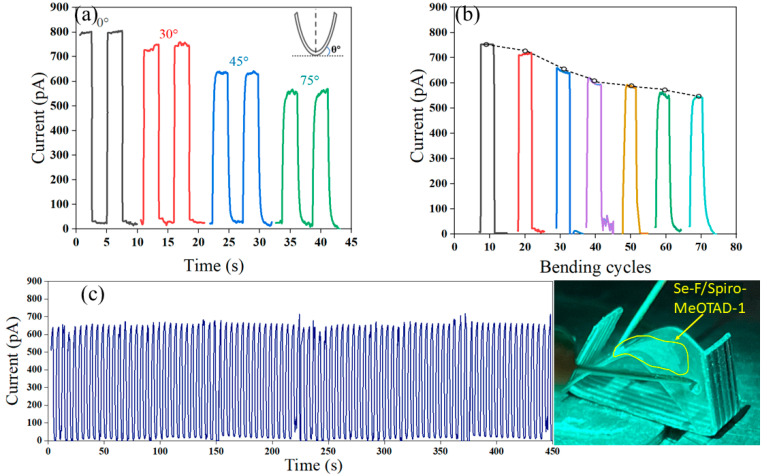
I-t curves of Se-F/Spiro-MeOTAD-1 flexible device at 410 nm with 0 V bias under different bending angles (**a**). (**b**) I-t curves after 0, 10, 20, 30, 40, 50, 60, and 70 cycles of bending at 30°, respectively. (**c**) Under 45° bending (inset photo) with periodic irradiation of ~450 s.

The Se-F/Spiro-MeOTAD-1 heterojunction flexible device was created using the same process after the Se-F was transferred to a PDMS substrate (Figure 9c). The I_light_ of the Se-F/Spiro-MeOTAD-1 PD decreases with greater bending degree in Figure 9a, and the I_light_ at 75° decreases to 73.4% of the original value without bending. The direct electron transport paths of Se-F and Spiro-MeOTAD-1 are reduced, leading to a decrease in I_light_ with increased bending angle. The response time also slows down as the bending degree increases. The response time of the Se-F/Spiro-MeOTAD-1 PD is 0.41 s/0.48 s at 75°, mainly due to the appearance of face defects in the Se lattice caused by bending with these defective grain boundaries acting as an obstruction to the drifting motion. The I_light_ of the Se-F/Spiro-MeOTAD-1 PD is maintained by about 76.3% after 70 cycles at 30°, further confirming the photodetection stability of the prepared flexible device under multiple bending conditions (Figure 9b). When bending induces mechanical strain on the Se-F film, this strain produces lattice defects and irreversible deformation in Se-F, resulting in a decrease in the photocurrent magnitude with an increasing number of bends. No significant photocurrent attenuation was observed after long-term cyclic irradiation of the flexible Se-F/Spiro-MeOTAD-1 device for approximately 450 s in Figure 9c. The results indicate that the device exhibits good stability under bending conditions.

## 4. Conclusions

In this paper, Spiro-MeOTAD-1 was prepared by using Zn(TFSI)_2_ and CNT:TiO_2_ as hybrid dopants to improve the stability of Spiro-MeOTAD. A hierarchical porous mesh Se-F film with good crystallinity was prepared using the solution method and annealing. The Se-F/Spiro-MeOTAD-1 heterojunction PD at 0 V bias exhibits a higher light current of 8.65 × 10^−10^ A, responsiveness of 108 mA W^−1^, and specific detectivity of 2.96 × 10^12^ Jones. The stability of photoelectric properties of the Se-F/Spiro-MeOTAD-1 PD are better than those of Se-F/Spiro-MeOTAD when placed for one month and at 140 °C. The light current of the Se-F/Spiro-MeOTAD-1 flexible PD can remain at 73.4% of its original value at 75° and 76.3% after being bent over 70 cycles, further demonstrating the photodetection stability under multiple bending conditions. After subjecting the device to repeated exposure to 410 nm light while bent for nearly 450 s, no considerable decrease in photocurrent is detected, suggesting the device maintains its stability in a bent configuration.

## Figures and Tables

**Table 1:** Comparison of optoelectronic device performances of Se-F/Spiro-MeOTAD-1 photodetectors among various devices.

Devices	Response Range (nm)	Bias (V)	On/Off	Responsivity (mA W^−1^)	Detectivity (Jones)	Rise/Fall Time (ms)	Ref.
Se/PANI	300–700	0	1000	120	3.78 × 10^11^	0.45/2.84	[24]
Se/PPy	300–700	0	220	70	2.21 × 10^11^	0.35/16.97	[24]
Se/PEDOT	300–700	0	50	5.5	1.76 × 10^10^	1.00/9.78	[24]
**Sb_2_Se_3_/GaN**	250–1250	0	19.2	1210	1.2 × 10^9^	0.669/0.672	[48]
**Sb_2_Se_3_ NTs**	400–1300	2	364	439	9.63 × 10^10^	27/27	[49]
**SnSe/Sb_2_Se_3_**	400–1200	0	-	0.24 × 10^−3^	-	60/134	[50]
**Sb_2_Se_3_/Cds**	400–1650	0	-	2.6	3.25 × 10^11^	0.009/0.011	[51]
**Se-F/Spiro-MeOTAD-1**	300–900	0	5000	109.8	2.98 × 10^12^	20/50	This work
**Se-F/Spiro-MeOTAD**	300–900	0	388	42.6	3.0 × 10^11^	25/50	This work

## Data Availability

Data are contained within the article.

## Supplementary Materials

The following supporting information can be downloaded at: https://www.mdpi.com/article/10.3390/nano15120916/s1, Figure S1. I-t curves of Se-F/Spiro-MeOTAD PD at 410 nm at 0.3 V bias after 100 cycles (a) and I-t curves of Se-F/Spiro-MeOTAD-1 PD at 410 nm at 0.25 V bias during 100 cycles (b). Figure S2. Optical image of Se-F/Spiro-MeOTAD PD (a) and Se-F/Spiro-MeOTAD-1 PD (b) with Ag electrodes and marked effective light area (S). Figure S3. The error ranges for the responsiveness of both Se-F/Spiro-MeOTAD PD and Se-F/Spiro-MeOTAD-1 PD samples. Figure S4.Cross-sectional view of Se-F/Spiro-MeOTAD-1.
