# Supplementary material for: CNT:TiO2-Doped Spiro-MeOTAD/Selenium Foam Heterojunction for High-Stability Self-Powered Broadband Photodetector
Source: Nanomaterials (Basel). 2025 Jun 12;15(12):916. doi: 10.3390/nano15120916 (PMC12195885; doi:10.3390/nano15120916)
Supplement: Supplementary file 1 [file nanomaterials-15-00916-s001.zip › nanomaterials-3648851-supplementary.pdf]

Supporting Information

CNT:TiO_2-Doped Spiro-MeOTAD/Selenium Foam Heterojunction for High-Stability Self-Powered Broadband Photodetector

Yuxin Huang [1], Pengfan Li [1], Xuewei Yu [1], Shiliang Feng [1], Yanfeng Jiang [1] and Pingping Yu [1*]

1 School of Integrated Circuits, Jiangnan University, Wuxi 214122, China
* Correspondence: pingpingyu@jiangnan.edu.cn

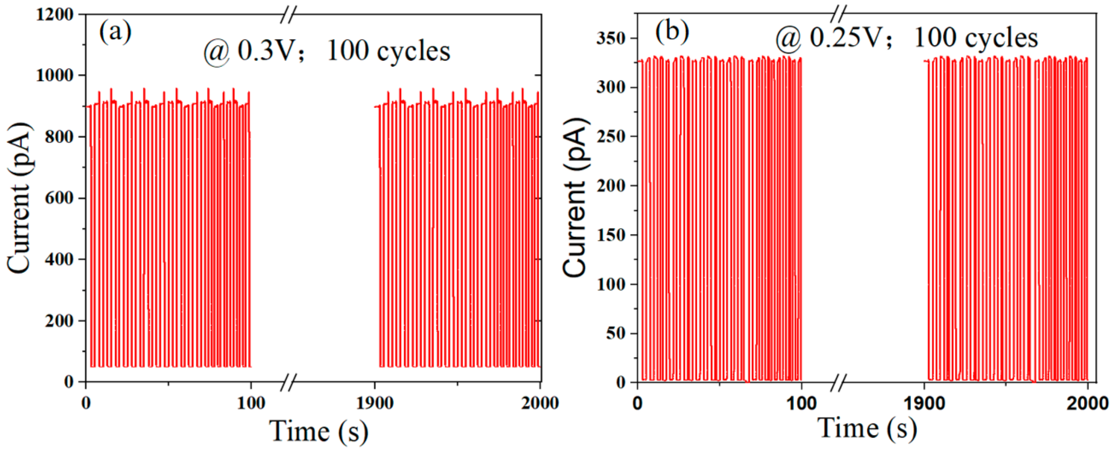

Figure S1. I-t curves of Se-F/Spiro-MeOTAD PD at 410 nm at 0.3 V bias after 100 cycles (a) and I-t curves of Se-F/Spiro-MeOTAD-1 PD at 410 nm at 0.25 V bias during 100 cycles (b).

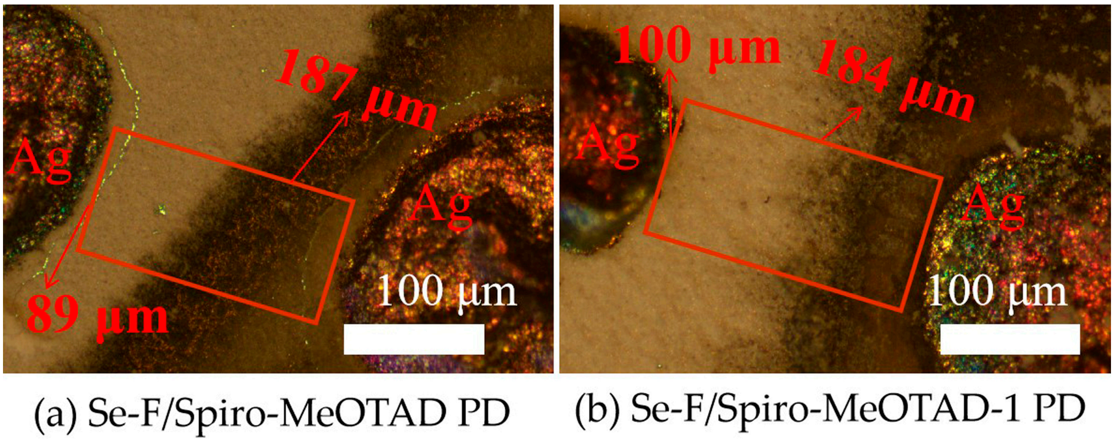

Figure S2. Optical image of Se-F/Spiro-MeOTAD PD (a) and Se-F/Spiro-MeOTAD-1 PD (b) with Ag electrodes and marked effective light area (S).

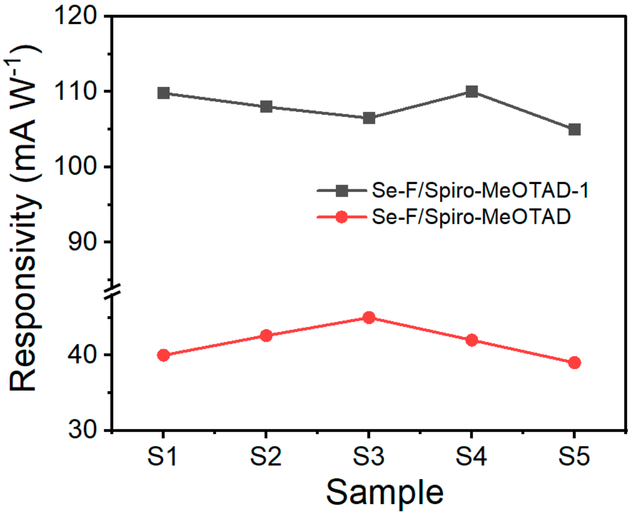

Figure S3. The error ranges for the responsiveness of both Se-F/Spiro-MeOTAD PD and Se-F/Spiro-MeOTAD-1 PD samples.

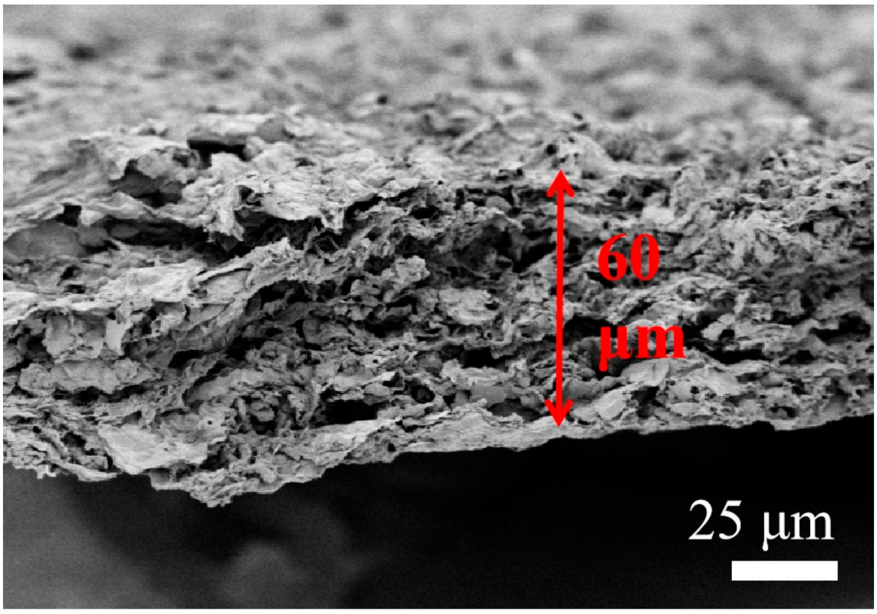

Figure S4.Cross-sectional view of Se-F/Spiro-MeOTAD-1.

Note 1. The special definitions of R, D*, and EQE are as follows:

Responsivity (R): Responsivity is defined as the ratio of the photocurrent generated per unit area of the detector to the incident light power, with units of A/W. The formulas are:

$$R_{\lambda} = \frac{I_{ph}}{PS} \quad (1\text{-}3)$$

$$D^{*} = \frac{R_{\lambda}}{\sqrt{2qJ_{d}}} \quad (1\text{-}4)$$

where P is the incoming light power, and I_{ph} is the difference between the light and dark currents ($I_{ph} = I_{light} - I_d$). S denotes the light-exposed region of heterojunction, which is 1.84×10^{-4} cm^2 for

Se-F/Spiro-MeOTAD and 1.67×10^{-4} cm^2 for Se-F/Spiro-MeOTAD-1, q is the charge, and J_d is the dark current density (I_d/S).

External Quantum Efficiency (EQE): External quantum efficiency is defined as the ratio of the charge carriers generated by the detected photons to the incident light. The formula is:

$$EQE=\frac{eI_{ph}}{P_{in}/hv}\times100\% \quad (1\text{-}5)$$

where e is the electron charge, Iph is the photocurrent, Pin is the incident light power, v is the frequency of light, and h is Planck's constant.

Note 2: The α for Spiro-MeOTAD-1 is 10.4×10^{-4} S cm^{-1}, which is much higher than that for spiro-MeOTAD of 5.83×10^{-4} S cm^{-1} (Ref. 42). The thickness of the Se-F/Spiro-MeOTAD-1 device is 60 μm observed from Figure S1. The C_{trap} was calculated by the following equations.

$$L_{eff} = \frac{1}{\alpha} ln(\frac{1}{1-\eta_{abs}})$$

$$C_{trap} = \frac{d}{L_{eff}}$$

where L_{eff} is the average propagation path length of photons (including multiple reflections/scattering) (2.53 μm), d is the physical thickness of the active layer (60 μm), α is the material absorption coefficient (10.4×10^{-4} S cm^{-1}), η_{abs} is the total absorptance of heterojunction (0.92), and n is the material refractive index (1.7). Therefore, the ideal C_{trap} is 11.56, but the real C_{trap} is 0.92. The device achieves only 7.96% of the theoretical limit that is necessary to optimize light-trapping structures.
